# Migration effects on the intestinal microbiota of Tibetans

**DOI:** 10.7717/peerj.12036

**Published:** 2021-10-18

**Authors:** Tian Liang, Fang Liu, Lifeng Ma, Zhiying Zhang, Lijun Liu, Tingting Huang, Jing Li, Wenxue Dong, Han Zhang, Yansong Li, Yaqiong Jiang, Weimin Ye, Su Bai, Longli Kang

**Affiliations:** 1 Key Laboratory for Molecular Genetic Mechanisms and Intervention Research on High Altitude Disease of Tibet Autonomous Region, School of Medicine, Xizang Minzu University, Xianyang, Shannxi Province, China; 2 Key Laboratory of High Altitude Environment and Genes Related to Diseases of Tibet Autonomous Region, School of Medicine, Xizang Minzu University, Xianyang, Shannxi Province, China; 3 Department of Medical Epidemiology and Biostatistics, Karolinska Institutet, Stockholm, Sweden; 4 Zashe Community Health Service Center, Lhasa, Tibet Autonomous Region, China

**Keywords:** Intestinal microbiota, Migration, Tibetan, Alpha diversity, Beta diversity

## Abstract

**Background:**

Diet, environment, and genomic context have a significant impact on humans’ intestinal microbiota. Moreover, migration may be accompanied by changes in human eating habits and living environment, which could, in turn, affect the intestinal microbiota. Located in southwestern China, Tibet has an average altitude of 4,000 meters and is known as the world’s roof. Xianyang is situated in the plains of central China, with an average altitude of about 400 meters.

**Methods:**

To understand the association between intestinal microbiota and population migration, we collected the fecal samples from 30 Tibetan women on the first day (as TI1st), six months (as TI2nd), and ten months (as TI3rd) following migration from Tibet to Xianyang. Fecal samples were collected from 29 individuals (belonging to the Han women) as a control. The dietary information of the Tibetan women and the Han women was gathered. We performed a 16S rRNA gene survey of the collected fecal samples using Illumina MiSeq sequencing.

**Results:**

Following the migration, the alpha and beta diversity of Tibetan women’s intestinal microbiota appeared unaffected. Linear discriminant analysis effect size (LEfSe) analysis showed that *Klebsiella*, *Blautia*, and *Veillonella* are potential biomarkers at TI1st, while Proteobacteria and Enterobacteriaceae were common in TI3rd. Finally, functional prediction by phylogenetic investigation of communities by reconstruction of unobserved states (PICRUSt) found no significant up-regulation or down-regulation gene pathway in the intestinal microbiota of Tibetan women after migration. The present study reveals that the higher stability in Tibetan women’s intestinal microbiota was less affected by the environment and diet, indicating that Tibetan women’s intestinal microbiota is relatively stable. The main limitations of the study were the small sample size and all volunteers were women.

## Introduction

The human intestinal microbiota plays a vital role in metabolism, digestion, immunity, and chronic diseases and has been suggested to co-evolve with the host (Tremaroli & Backhed, 2012; Turnbaugh et al., 2006). Dietary habits, living environment, migration, and genetic background can influence the intestinal microbiota (Kwok et al., 2014; Blaser & Falkow, 2009; De Filippo et al., 2010; Gomez et al., 2016; Obregon-Tito et al., 2015). Vangay et al. reported that human intestinal microbiota composition changes after migrations or travel (DuPont, 2016; Keohane et al., 2020; Vangay et al., 2018; Waldron, 2015). However, an individual’s unique genetic background can maintain intestinal microbiota stability and ensure resistance to external interference (Mullaney et al., 2019).

Tibet, located on the Qinghai-Tibet Plateau in China, has an average altitude of >4 km, an annual average temperature of 5.2 °C, low oxygen content, and intense ultraviolet rays (Cuo & Zhang, 2017; Li & Zhao, 2015; Aldenderfer, 2011). Native Tibetans are genetically adapted to the low-oxygen and low-pressure environment (Li et al., 2016c; Lorenzo et al., 2014; Wang et al., 2016; Xu et al., 2015; Yang et al., 2017). Previous studies have revealed phenotypic features in native Tibetans associated with plateau adaptation, including low hemoglobin concentration, low pulmonary arterial pressure, enhanced athletic endurance, and low incidence of chronic high altitude disease (Beall et al., 2010; Ge et al., 2011; Kang et al., 2013; Kang et al., 2016; Lu et al., 2012; Simonson et al., 2010). Furthermore, current studies have found that hypoxia influences intestinal microbiota diversity and richness (Jia et al., 2020).

Several studies have shown that plateau animals and humans have a higher diversity of intestinal microbiota, a more stable microbial network structure, and can better adapt to extremely harsh environments (Jia et al., 2020; Zeng et al., 2020). Compared to the plains’ Han populations, Tibetans’ intestinal microbiota is significantly different in composition and function (Li & Zhao, 2015). The exceptional environment of Tibet and the genetic background of Tibetans could lead to the unique intestinal microbiota (Li & Zhao, 2015). The diet, culture, and lifestyle of Tibetans are significantly different from those of the Han Chinese. Specifically, Tibetans consume more meat and less fresh fruits and vegetables (Dan et al., 2013).

It has been demonstrated that when the population migrates, the intestinal microbiota changes significantly, and the incidence of metabolic diseases increased (Guo et al., 2019; Vangay et al., 2018). In the past several decades, many Tibetans have migrated to Xianyang, Shaanxi Province, a plain region in central China. Xianyang has an average altitude of approximately 400 m and is rich in various vegetables that are rare in Tibet. Thus, a plant—animal-balanced dietary pattern, rich in high-starch foods such as noodles and rice, is dominant in Xianyang (Huang et al., 2019). To date, the relationship between the intestinal microbiota of Tibetan women and migration has not been reported. Therefore, our primary aim was to explore whether Tibetan women’s intestinal microbiota can resist the changes in dietary habits and living environment elicited by migration.

## Materials & Methods

### Sample collection

Thirty native Tibetan women volunteers who had migrated from five regions of Tibet (Chamdo, Lhasa, Naqu, Shannan, and Shigatse) (average altitude of approximately 4000 m) were recruited from the Xizang Minzu University in Xianyang (average altitude of approximately 400 m). The volunteers’ living environment was at an altitude of 3500–4500 m, the migration distance was greater than 3000 km, and the maximum altitude span was greater than 4000 m. Also, twenty-nine Han women volunteers from Xianyang, Shaanxi Province, were enrolled as controls. Their living environment was at an altitude of 400 m. All volunteers participating in the experiment were women, and the information of the volunteers about age, gender, height, weight, body mass index (BMI), ethnicity, and dietary questionnaires were collected (Tables 1–2). The dietary habits of Tibetan women we collected were classified into two parts, (1) the dietary habits of Tibetan women in Tibet (TI1st) and (2) the dietary habits of Tibetan women in Xianyang (TI2nd and TI3rd).

Fresh stool samples were collected from the Tibetan women three times from September 2016 to June 2017: the first day arrival in Xianyang (TI1st), six months (TI2nd), and ten months (TI3rd) after arrival. A total of 90 stool samples were collected (Tables 3–4). Only one batch of fresh stool samples was collected from the Han women during the enrollment, named HI. The inclusion criteria for the samples were: (i) no intestinal diseases; (ii) no antibiotic use within three months before sample collection. Approximately 5 g fecal matter was collected from each volunteer and stored in 10-mL sterile tubes, at −80 °C until further use. An informed consent form was signed by all participants declaring that they fully understood the purpose of this research. The Ethics Committee of Xizang Minzu University approved the study (ID: 201601), and written permission from all participants was submitted. The present study strictly followed the standard biosecurity and safety procedures of Xizang Minzu University.

### Bacterial DNA extraction and PCR amplification

The TIANamp Stool DNA Kit (Shanghai, China) was used to extract DNA from stool samples and perform enzymatic lysis and bead-beating. The indexed libraries targeting the hypervariable V3–V4 region of the 16S rRNA gene were amplified using the universal 341F (5′-CCTACGGGNGGCWGCAG-3′) and 805R (5′- GACTACHVGGGTATCTAATCC-3′) primers. PCR amplifications were performed in a 10 µl reaction, including 0.2 µL of each primer (10 µM), 3 µl of microbial DNA, 1 µL 10 × buffer, 0.8 µL dNTPs (25 mM), 0.2 µL Toptaq DNA Polymerase, and 4.6 µL ddH2O. Thermal cycling consisted of initial denaturation at 94 °C for 2 min, followed by 25 cycles consisting of denaturation at 94 °C for 30 s, annealing at 55 °C for 30 s, elongation at 72 °C for 1 min, and final elongation at 72 °C for 10 min.

**Table 1:** Volunteer information.

	Han	Tibetan 1
Age (years ± SD)	19 ± 1	19 ± 1
Height	165.07 ± 8.52	162.10 ± 7.97
Weight	55.83 ± 11.85	56.85 ± 10.62
BMI	20.77 ± 4.40	21.59 ± 3.26
Gender (male/the total number of people)	0%	0%
Gender (female/the total number of people)	100%	100%

**Table 2:** Dietary information.

	Han	Tibetan 1 (pre-migration)	Tibetan 2 and 3 (post-migration)
Main animal food	Pork, chicken, egg	Yak meat, mutton	Pork, chicken
Frequency of meat consumption	Once per day	2–3 times per day	Once per day
Main vegetable food	Various vegetables	Chinese cabbage, potato, tomato	Various vegetables
Frequency of vegetable consumption	2–3 times per day	Once per day	2–3 times per day
Main fruits food	Various fruits	Apple, banana, orange	Various fruits
Frequency of fruits consumption	Once per day	Once per 2–3 days	Once per day
Staple food	Rice, noodles	Zanba, butter tea	Rice, noodles
Frequency of Staple food consumption	2–3 times per day	2–3 times per day	2–3 times per day

**Table 3:** Geographic information.

Region	No. of individuals	Ethnic group	Mean altitude	Longitude and latitude
Chamdo	7	Tibet	3500	28°5′–32°6′N, 93°6′–99°2′E
Lhasa	11	Tibet	3650	29°36′N, 91°06′E
Nagqu	1	Tibet	4500	29°55′–36°30′N, 83°55′–95°5′E
Shannan	2	Tibet	3700	27°08′–29°47′N, 90°14′–94°22′E
Shigatse	9	Tibet	4000	27°13′–31°49′N, 82°1′–90°20′E
Xianyang	29	Han	400	108°70′N, 34°33′E

**Table 4:** Sample information.

Sample size		TI1st/HI1st (one day)	TI2nd (six months)	TI3rd (ten months)	Total
Tibetan fecal samples		30	30	30	90
Han fecal samples		29	0	0	29

### DNA library construction and high-throughput sequencing

PCR products were separated by 2% agarose gel electrophoresis, purified using AMPure XP beads (Beckman Coulter, USA), and quantitated using a Quantus™ fluorometer (Promega, USA). Ultimately, the DNA library was obtained using a NEXTFLEX Rapid DNA-Seq Kit (Illumina), merged into equimolar concentrations, and sequenced using an Illumina MiSeq platform with a 2 ×250 paired-end protocol. The raw sequence data were deposited in the Genome Sequence Archive at the Data Center, Beijing Institute of Genomics (BIG), Chinese Academy of Sciences, under accession numbers CRA002412 and CRA002780. The shared URL is http://bigd.big.ac.cn.

### Processing of the sequencing data

Raw FASTQ files were demultiplexed, quality-filtered by Trimmomatic v0.39 (Bolger, Lohse & Usadel, 2014), and merged by FLASH v1.2.11 (Magoc & Salzberg, 2011) using the following criteria: (1) The reads were truncated at any site receiving an average quality score less than 20 over a 50 bp sliding window; (2) Primers were matched allowing two nucleotide mismatches, and reads containing ambiguous bases were removed; (3) Sequences with overlaps longer than 10 bp were merged according to their overlap sequence. Operational taxonomic units (OTUs) were clustered with a 97% similarity cutoff using UPARSE v7.1 (http://drive5.com/uparse/, Edgar, 2013), and chimeric sequences were identified and removed using UCHIME v4.1 (Edgar et al., 2011). The taxonomy of each 16S rRNA gene sequence was analyzed by the RDP Classifier algorithm v2.2 (https://rdp.cme.msu.edu/tutorials/classifier/classifer_cover_page.html, Lu et al., 2020) against the Silva (SSU123) 16S rRNA database using a confidence threshold of 80%.

### Bioinformatics and statistical analysis

Species accumulation curves were drawn using the R software v3.6.2 (‘vegan’ package). The Chao 1, Observed species, Simpson, Shannon indexes were measured using the Mothur software v1.41.1 (Schloss et al., 2009). Different groups were analyzed using the weighted and unweighted UniFrac distance of principal coordinates analysis (PCoA) and permutational multivariate analysis of variance (PERMANOVA) (Kelly et al., 2002; Lozupone & Knight, 2005). Linear discriminant analysis effect size (LEfSe software v1.0, (Segata et al., 2011) was carried out to find biomarkers with significant differences between different groups, and the LDA score was set to 2.0. The phylogenetic investigation of communities by reconstruction of unobserved states (PICRUSt, v1.1.1) software (Langille et al., 2013) was employed to predict the potential functions of the intestinal microbiota and further analyze them in the context of the Kyoto Encyclopedia of Genes and Genomes (KEGG) databases (Kanehisa & Goto, 2000). The enterotype analysis was performed by the Enterotypes (http://enterotypes.org, Costea et al., 2018). Costea’s research suggests that human intestinal types are mainly composed of enterotype_Firmicute (ET_F), enterotype_*Bacteroide* s (ET_B), and enterotype_*Prevotella* (ET_P). Firmicutes, *Bacteroides* and *Prevotella* were the driving genus of ET_F, ET_B, and ET_P, respectively (Costea et al., 2018). A Chi-square test was used to verify the difference in bowel type between different groups. The student’s *t*-test compared the demographic information of the Han and Tibetan women. The Wilcoxon rank-sum test was employed to compare the relative abundance of predominant bacteria and functional prediction between HI and TI1st groups. The Wilcoxon signed-rank test was used to test the significant differences in the intestinal microbiota of Tibetan women at two different time points, and the Friedman test was used to test the differences in the intestinal microbiota of Tibetan women at three different time points. *P*-values were subjected to false discovery rate (FDR) correction; a *q* < 0.05 was considered statistically significant.

## Results

### Eating habits of Tibetan and Han women

A total of 119 stool samples (90 samples from 30 Tibetan women and 29 samples from 29 Han women) were sequenced using the Illumina Miseq platform, with 9,313,735 sequences being obtained. Following quality control, a total of 8,566,092 sequences were obtained, with an average of 71,984 sequences (43,256–97,150) per sample, and a total of 34,745 independent OTUs were obtained after 97% clustering (Figs. S1A–S1D). Age, height, weight, and BMI showed no significant differences between Han and Tibetan women (Table 1). We found that the diet of Tibetan women in Tibet (TI1st group) was mainly meat with a low intake of vegetables and fruits. The Han women (HI group) diet was balanced with moderate consumption of meat, vegetables, and fruits. After Tibetan women migrated to Xianyang (TI2 and TI3rd groups), their eating habits changed, the meat intake decreased, and the intake of vegetables and fruits increased (Table 2).

### Comparison of intestinal microbiota between Tibetan and Han women

**Figure 1:**
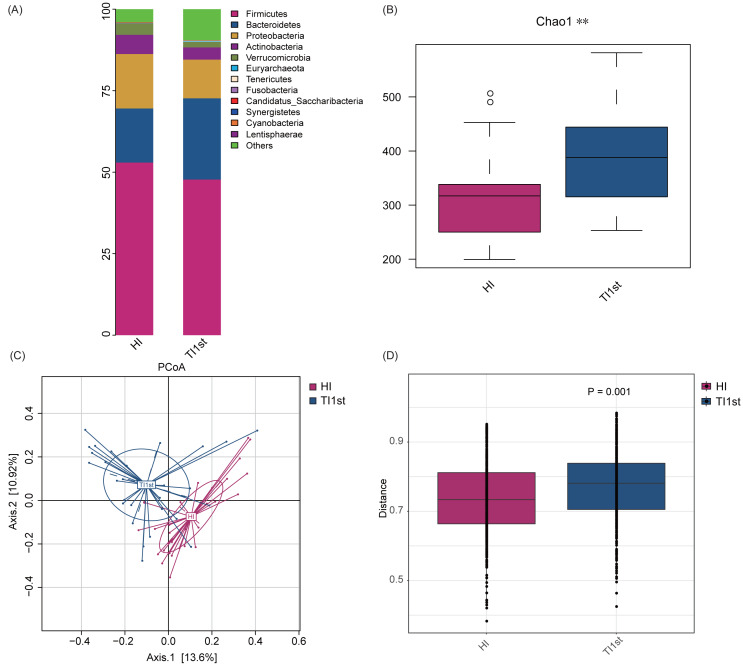
Comparison of alpha and beta diversity of intestinal microbiota between Tibetans and Han women. (A) Composition of bacterial communities. The alpha diversity (B) and beta diversity (weighted UniFrac distance) (C–D) of the intestinal microbiota of Tibetan women are significantly higher than that of the Han women, ** *P* < 0.01.

Firmicutes, Bacteroidetes, Proteobacteria, Actinobacteria, and Verrucomicrobia constituted the five most dominant bacterial phyla in the intestinal microbiota of Han and Tibetan women (relative abundance >0.1%, Fig. 1A). The Chao 1, Observed species, and Simpson and Shannon indexes were used to assess the microbial community’s alpha diversity. The HI group’s alpha diversity was significantly lower than that of the TI1st group (*P* < 0.05, Fig. 1B, Figs. S2A–S2C). The beta diversity weighted and unweighted UniFrac distances based on PCoA analysis showed that the beta diversity of the TI1st group was significantly higher than that of the HI group (R^2^ = 0.06, *P* < 0.05, Figs. 1C–1D, Figs. S2D–S2E).

**Figure 2:**
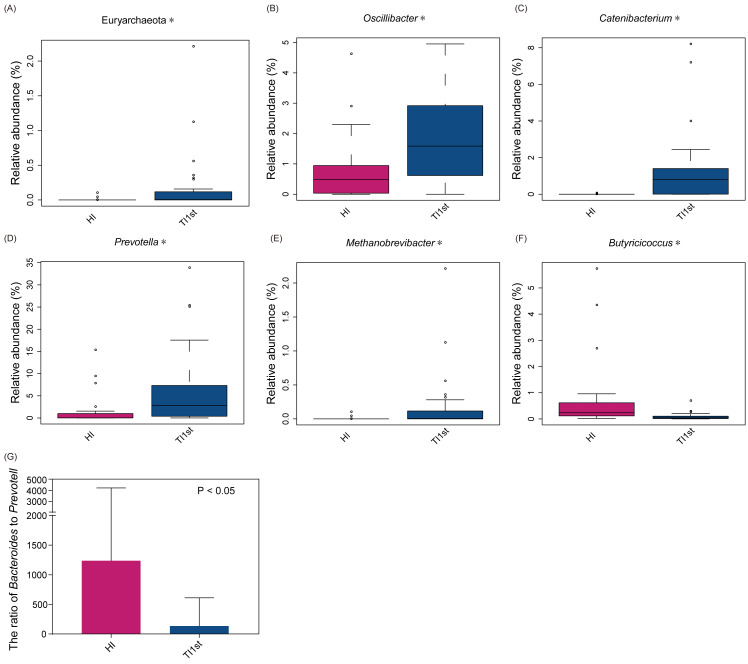
Comparison of bacterial abundance between Tibetan and Han women at the phylum level (A) and genus level (B–F). Comparison of bacterial abundance between Tibetan and Han women at the phylum level (A) and genus level (B–F). (G) The ratio of *Bacteroides* to *Prevotella* in the HI group was significantly higher than that in the TI1st group, * *P* < 0.05.

The dominant bacteria in Tibetan women was different from that of the Han women. At the phylum level, the relative abundance of Euryarchaeota in the TI1st group was significantly higher than in the HI group (relative abundance>0.1%, *P* < 0.05, Fig. 2A). Meanwhile, the relative abundance levels of *Oscillibacter*, *Catenibacterium*, *Prevotella,* and *Methanobrevibacter* were higher in the TI1st group at the genus level (*P* < 0.05, Figs. 2B–2E). In contrast, the relative abundance levels of *Butyricicoccus* were higher in the HI group (relative abundance>0.1%, *P* < 0.05, Fig. 2F). Comparison of *Bacteroides* and *Prevotella* ratios (B/P) showed that the B/P significantly higher in the HI group than in the TI1st group (relative abundance>0.1%, *P* < 0.05, Fig. 2G).

**Figure 3:**
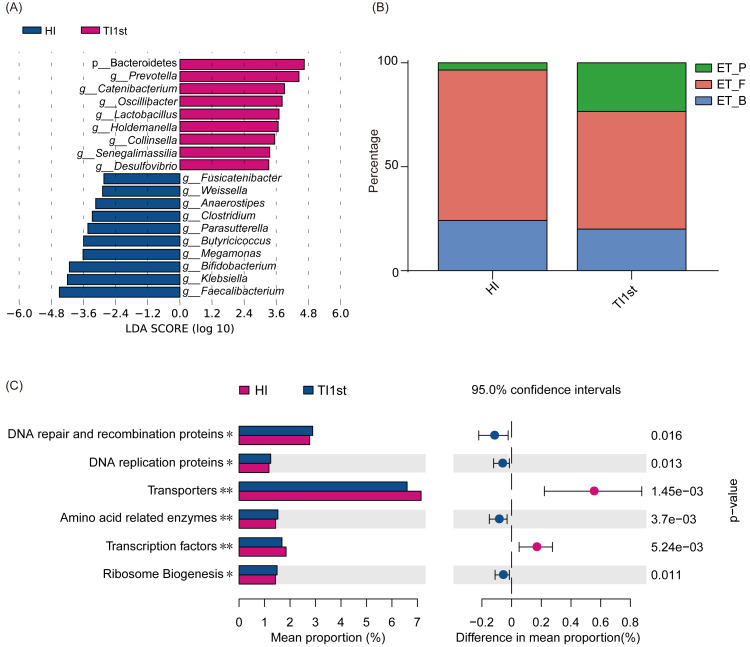
Comparison of biomarkers, enterotypes, and KEGG pathways between Tibetan and Han women. (A) LEfSe analysis results for the HI and TI1st groups; LDA score was set to 2.0. (B) Comparison of enterotype between HI and TI1st groups, *P* > 0.05, *χ*^2^ = 4.97. (C) The significantly different KEGG pathways between the HI and TI1st groups, * *P* < 0.05, ** *P* < 0.01.

LEfSe analysis was performed to elucidate differential biomarkers between the two groups (HI and TI1st) (Fig. 3A). Biomarkers in TI1st group were p__Bacteroidetes, *g*__*Prevotella*, *g*__*Catenibacterium*, *g*__*Oscillibacter*, *g*__*Lactobacillus*, *g*__*Holdemanella*, *g*__*Collinsella*, *g*__*Senegalimassilia*, *g*__*Desulfovibrio.* While, the biomarkers in HI group were *g*__*Fusicatenibacter*, *g*__*Weissella*, *g*__*Anaerostipes*, *g*__*Clostridium*, *g*__*Parasutterella*, *g*__*Butyricicoccus*, *g*__*Megamonas*, *g*__*Bifidobacterium*, *g*__*Klebsiella* and *g*__*Faecalibacterium* (LDA >2, *P* < 0.05).

Costea’s research suggests that human intestinal types are mainly composed of enterotype_Firmicute (ET_F), enterotype_*Bacteroide* s (ET_B), and enterotype_*Prevotella* (ET_P). Firmicutes, *Bacteroides,* and *Prevotella* were the driving genus of ET_F, ET_B, and ET_P, respectively (Costea et al., 2018). Our research found that the proportions of ET_F (HI: 72%, TI1st: 60%) and ET_B (HI: 23%, TI1st: 20%) in the HI and TI1st groups were similar. Also, although the proportion of ET_P (HI: 5%, TI1st: 20%) in the TI1st group was higher than that of the HI group, no statistically significant difference was observed (*P* > 0.05, Fig. 3B). Gene function prediction based on the KEGG database found that DNA repair and recombination proteins, DNA replication proteins pathways, amino acid-related enzymes, and ribosome biogenesis were up-regulated in the TI1st group, while the transcription factors and transporters pathways were up-regulated in the HI group (*P* < 0.05, Fig. 3C).

### Alterations in Tibetan women intestinal microbiota following migration to Xianyang

**Figure 4:**
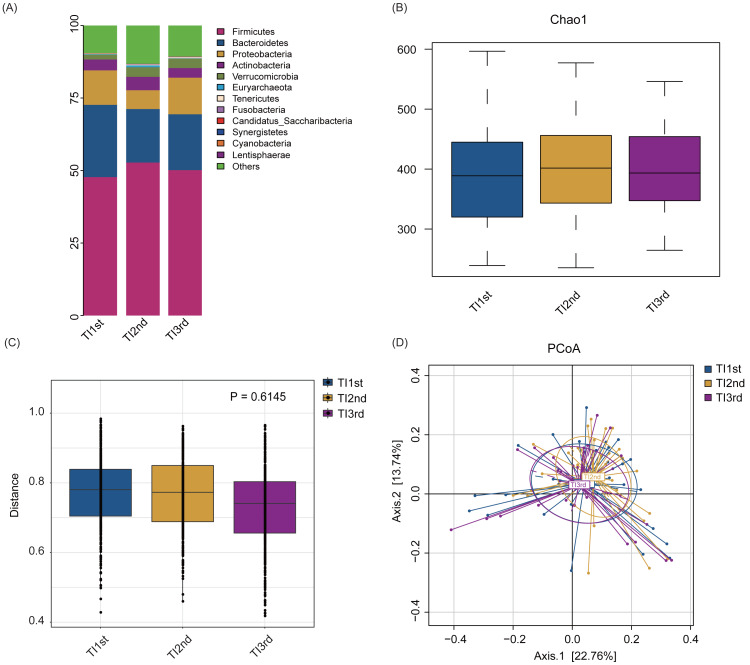
Comparison of alpha and beta diversity of intestinal microbiota among TI1st, TI2nd, and TI3rd groups. (A) Composition of bacterial communities. No significant differences in the alpha diversity (B) and beta diversity (weighted UniFrac distance) (C–D) of the intestinal microbiota of Tibetan women compared with the Han women, *P* > 0.05.

Firmicutes, Bacteroidetes, Proteobacteria, Actinobacteria, Verrucomicrobia, and Euryarchaeota constituted the six most dominant bacterial phyla in the TI1st, TI2nd, and TI3rd groups (relative abundance >0.1%, Fig. 4A). There was no significant difference in alpha diversity among TI1st, TI2nd, and TI3rd groups (Fig. 4B, Figs. S3A–S3C, *P* > 0.05). Figures 4C–4D shows no significant differences in the structure of microbial communities between TI1st, TI2nd, and TI3rd groups (PERMANOVA, R ^2^ = 0.0207, *P* = 0.6217, Figs. S3D–S3E).

**Figure 5:**
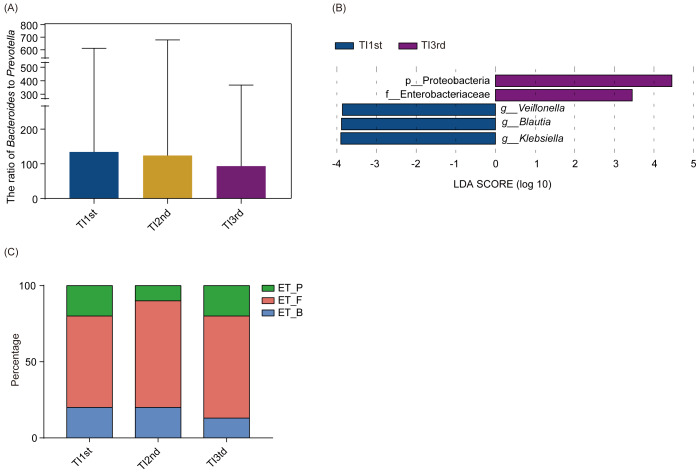
Comparison of the ratio of *Bacteroides* to *Prevotella*, biomarkers, and enterotypes among TI1st, TI2nd, and TI3rd groups. (A) Comparison of the *Bacteroides* to *Prevotella* ratio among TI1st, TI2nd, and TI3rd groups, *P* > 0.05. (B) LEfSe analysis results for the TI1st, TI2nd, and TI3rd groups; LDA score was set to 2.0. (C) Comparison of enterotype among TI1st, TI2nd and TI3rd groups, *P* > 0.05, *χ*^2^ = 0.68.

There was no significant difference in phyla and genera abundances among TI1st, TI2nd, and TI3rd groups (relative abundance>0.1%, *P* > 0.05, Figs. S4A–S4B). No significant difference was found in the B/P of the TI1st, TI2nd, and TI3rd groups (*P* > 0.05, Fig. 5A). LEfSe analysis reflected significant differences in five bacterial taxa among TI1st, TI2nd, and TI3rd (Fig. 5B). In TI1st, *g_Veillonella*, *g_Blautia*, and *g_Klebsiella* were more abundant. p_Proteobacteria and *un_f_Enterobacteriaceae* were highly enriched in the TI3rd group (LDA >2, *P* < 0.05). No biomarker was found in the TI2nd group. Results show that the proportions of ET_F (TI1st: 60%, TI2nd: 70%, TI3rd: 67%) and ET_P (TI1st: 20%, TI2nd: 10%, TI3rd: 20%) in the TI1st, TI2nd, and TI3rd groups were similar (Fig. 5C). Although the proportion of ET_B in the Tibetan women intestinal microbiota continues to decrease with time, and the proportion of ET_B in the TI3rd group was lower than that in the TI1st and TI2nd groups (TI1st: 20%, TI2nd: 20%, TI3rd: 13%, Fig. 5C), however, the reduction was not statistically significant (*P* > 0.05).

**Figure 6:**
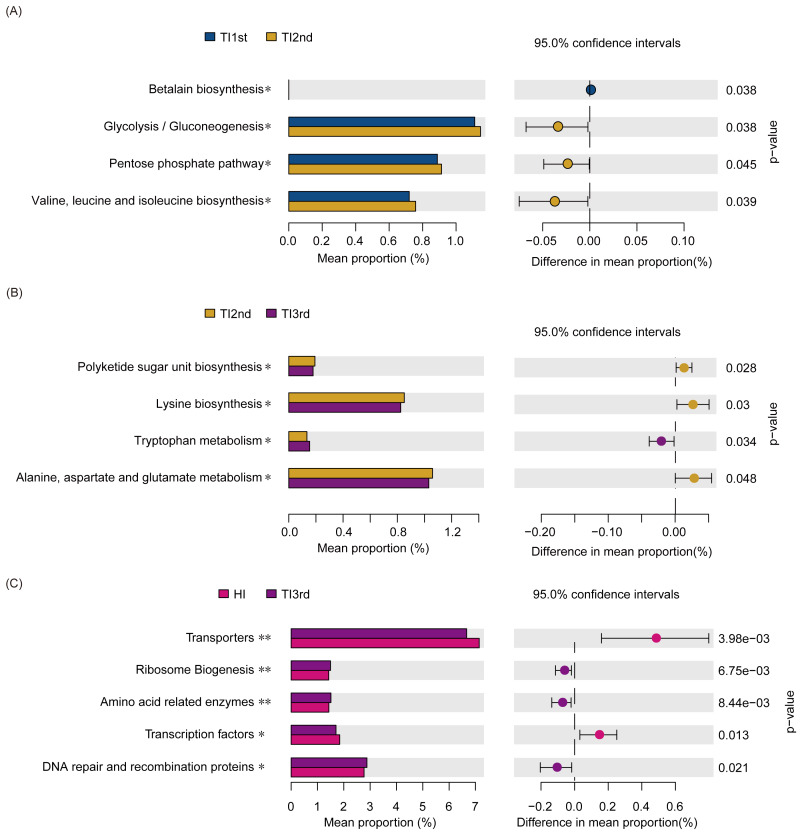
Function prediction of intestinal microbiota among TI1st, TI2nd, and TI3rd groups. (A) The significantly different KEGG pathways between the TI1st and TI2nd groups (A), TI2nd and TI3rd groups (B), TI3rd and HI groups (A), * *P* < 0.05.

Compared with the TI1st group, the betalain biosynthesis metabolism pathways were down-regulated in the TI2nd group, while the pentose phosphate, glycolysis/gluconeogenesis, and some amino acid (valine, leucine, and isoleucine) metabolism pathways were up-regulated (Fig. 6A). Compared with the TI2nd group, the amino acid (alanine, aspartate, and glutamate) metabolism pathways, polyketide sugar unit, and lysine biosynthesis pathways were down-regulated in the TI3rd group, while the tryptophan metabolism pathway was up-regulated (Fig. 6B). However, there was no significant difference in gene pathways between TI1st and TI3rd groups (Table S1), and there was no significant difference among TI1st, TI2nd, and TI3rd groups (Fig. S4C). We further compared the gene pathways of the TI3rd and HI groups, and the results showed that the most abundant gene pathways in the TI3rd group were DNA repair and recombination proteins, amino acid-related enzymes, and ribosome biogenesis pathways. In contrast, the most abundant gene pathway in the HI group were transcription factors and transporters pathways (Fig. 6C).

## Discussion

Living environment, eating habits, altitude, migration, and genetic background play a significant role in shaping the microbiome (Brooks et al., 2018; Deschasaux et al., 2018; Gill et al., 2018; Dall et al., 2019; Li & Zhao, 2015; Mullaney et al., 2019). Microbes were affected by extreme environments’ enormous selection pressure (high altitude, low oxygen, and low temperature) (Jia et al., 2020; Li & Zhao, 2015; Li et al., 2019). The microbiota’s stability increases with alpha diversity and, therefore, is less disturbed by the external environment (Bannar-Martin et al., 2018; Louca et al., 2018). The Tibetans’ intestinal microbiota has high diversity and stability to adapt to extreme environments and maintain a normal physiological metabolism (Jia et al., 2020; Li & Zhao, 2015). Migration and travel usually cause changes in the structure of human intestinal microbiota, affecting digestion and absorption, contributing to a high incidence of metabolic diseases (Dall et al., 2019; Vangay et al., 2018). We investigated whether the relatively stable intestinal microbiota of Tibetan women could also resist the effects of migration. Therefore, in the present study, Illumina Miseq sequencing was performed to characterize the structural and functional changes in the intestinal microbiota of Tibetan women (high-altitude areas) following migration to Xianyang (plains) using Han women as a control. However, we admit that there were some limitations in this study, including small sample size, and all volunteers were women.

### Differences in the diversity of intestinal microbiota between Han and Tibetan women

Diet, environment, and genetics are important factors that affect the intestinal microbiota (Gomez et al., 2016; Kwok et al., 2014). In the current study, the alpha and beta diversity of the intestinal microbiota of Tibetan women is significantly higher than that of Han women. The community structure of the intestinal microbiota of Tibetan women is significantly different from that of Han women. The host intestinal microbiota’s alpha and beta diversity may be affected by differences in dietary habits (Portune et al., 2017). Tibetans and Mongolians have similar eating habits. The Mongolian population diet includes meat, and its alpha diversity is much higher than that of the Han, who have a balanced diet (Liu et al., 2016). Moreover, there are also significant differences in the bacterial community structure of the Mongolian and Han populations (Liu et al., 2016). The beta diversity of the host’s intestinal microbiota will be affected by dietary patterns and composition. Animal experiments have shown that the beta diversity of the intestinal microbiota of plateau pikas (*Ochotona curzoniae*) is affected by dietary components’ beta diversity (Li et al., 2016a; Shikany et al., 2019). In conclusion, eating habits may be one of the factors that cause the differences in the alpha and beta diversity of the intestinal microbiota of Tibetan and Han women.

Furthermore, hypoxia and low temperature influence intestinal microbiota’s alpha and beta diversity (Jia et al., 2020). Intermittent hypoxia can increase the mouse intestinal microbiota’s alpha diversity and induce changes in the community structure (Moreno-Indias et al., 2015). The alpha diversity of mice’s intestinal microbiota at 4 °C is significantly higher than that of mice at 20 °C (Li et al., 2019a). The structure of human intestinal microbiota is also affected by low temperature. The intestinal microbiota’s alpha diversity of high-altitude Tibetans is significantly higher than that of low-altitude Tibetans (Lan et al., 2017). Altitude also induces significant differences in their bacterial communities’ structure (Lan et al., 2017). The effects of altitude on bacterial alpha diversity and communities’ structure has been confirmed in animal experiments (Wu et al., 2020). A skin microbiota study found that human skin microbiota’s beta diversity increases with altitude (Li et al., 2019b). In summary, hypoxia, low temperature, and high altitude may increase the intestinal alpha diversity and change the community structure.

The host’s genetic background could lead to differences in the intestinal microbiota’s structure and function (Mullaney et al., 2019). There are significant differences in the diversity and community structure of the intestinal microbiota of different ethnic groups (Zhang et al., 2015). The alpha and beta diversity of the Tibetan population’s intestinal microbiota is significantly different from that of the Han population (Zhang et al., 2015; Nakayama, Zhang & Lee, 2017), which is consistent with our research. Taken together, we speculate that the differences in alpha and beta diversity of the intestinal microbiota of the Tibetan and Han women due to dietary habits, living environment, and genetic background factors.

### Variations in the alpha and beta diversity of Tibetan women intestinal microbiota following migration

Dietary habits and living environment can change with migration, which are the two most essential factors affecting intestinal microbiota (Gomez et al., 2016; Rothschild et al., 2018). In the present study, the alpha and beta diversities of the intestinal microbiota of Tibetan women did not change significantly (TI1st, TI2nd, TI3rd) following migration and changing diet and environment. The alpha diversity of the intestinal microbiota of Tibetan women ten months (TI3rd) after migrated to Xianyang was still higher than that of Han women (HI). There are many short-chains fatty acid-producing bacteria in the intestine of Tibetans (Li & Zhao, 2015; Li et al., 2016b), which can help maintain the intestinal stability microbiota, and our research had similar findings. Short-chain fatty acids could activate the transcription factor HIF-1 in intestinal epithelial cells, alleviate intestinal inflammation, and maintain high stability and diversity of intestinal microbiota (Chen & Stappenbeck, 2019; Fachi et al., 2019; Kriss et al., 2018). The plateau environment of Tibet and the unique genetic background of the Tibetan women might also contribute to maintaining a high diversity and high stability of the intestinal microbiota. Additionally, the limited sample size and sample representativeness of Tibetan women might bias the diversity results. Therefore, the alpha and beta diversity of the intestinal microbiota did not change after the Tibetan women migrated to Xianyang, which may be caused by genetic factors.

### Differences in the bacterial abundance of intestinal microbiota between Han and Tibetan women

In this study, we identified *Euryarchaeota*, *Prevotella*, *Catenibacterium*, *Oscillibacter*, *Lactobacillus*, *Holdemanella*, *Collinsella*, *Methanobrevibacter,* and *Desulfovibrio* in higher abundance in the intestinal microbiota of the Tibetan women. First, we discover that the abundance of short-chain fatty acid-producing bacteria in the intestinal microbiota of Tibetan women, such as *Prevotella*, *Lactobacillus*, and *Holdemanella* (Zhang et al., 2015; Zagato et al., 2020). Li’s research also demonstrated that the abundance of short-chain fatty acid-producing bacteria in Tibetans’ intestines is higher than that of the Han population (Li & Zhao, 2015; Li et al., 2016b). This might be due to short-chain fatty acids can help Tibetans regulate blood pressure and pulmonary hypertension and help them adapt to the high altitude environment (Pluznick, 2017). *Catenibacterium*, *Collinsella,* and *Oscillibacter* may be unique biomarkers of Tibetan intestinal microbiota (Li & Zhao, 2015). *Collinsella* is strongly associated with obesity and atherosclerosis and is also closely related in the high incidence of cardiovascular disease in Mongolians (Jung et al., 2016; Astbury et al., 2020). Due to excessive fat intake, individuals can experience an increased abundance of *Oscillibacter* and *Catenibacterium* (Pujo et al., 2020).

In this study, we also found a higher abundance of the archaea Euryarchaeota and *Methanobrevibacter* in the intestinal microbiota of Tibetan women. This could be due to the syntrophic relationship between short-chain fatty acid-producing bacteria and archaea (Ruaud et al., 2020). Hydrogen, a metabolite of short-chain fatty acid-producing bacteria, can be used as a fermentation substrate for archaea (Ruaud et al., 2020). The process by which archaea consume hydrogen to generate methane inhibits the fermentation pathway of short-chain fatty acids, thereby increasing the production of short-chain fatty acids (Ruaud et al., 2020). Archaea consumes hydrogen to generate methane and inhibits the fermentation pathway of short-chain fatty acids, which leads to an increase in the production of short-chain fatty acids (Ruaud et al., 2020). Our studies demonstrate that the abundance of *Desulfovibrio* in Tibetan women’s intestines is higher, which may be caused by the low-temperature environment (Ziętak et al., 2016). After mice are exposed to a cold environment, the abundance of *Desulfovibrio* in their intestinal microbiota increases significantly (Ziętak et al., 2016).

Our research found that the abundance of *Weissella*, *Clostridium*, *Butyricicoccus*, *Parasutterella*, and *Klebsiella* in the Han women’s intestinal microbiota is higher than in Tibetan women. *Weissella* enhances the immune response of the host by increasing the activity of natural killer cells (Lee et al., 2018). We also found short-chain fatty acid-producing bacteria in the intestines of the Han women, such as *Clostridium* and *Butyricicoccus* (Lee et al., 2020). The abundance of opportunistic pathogens in the intestine of patients with Crohn’s disease is higher, while the abundance of short-chain fatty acid-producing bacteria is significantly reduced (Wang et al., 2018). The abundance of opportunistic pathogens is negatively correlated with the abundance of short-chain fatty acid-producing bacteria (Gong et al., 2019; Zeng et al., 2019). We speculate that the high abundance of potentially pathogenic bacteria in the intestines of the Han women inhibited short-chain fatty acid-producing bacteria growth. Therefore, compared with Tibetan women, there are fewer types of short-chain fatty acid-producing bacteria in the intestines of Han women.

### Variations in the bacterial abundance of Tibetan women intestinal microbiota following migration

The LEfSe software elucidated statistically significant differences in biomarkers among the TI1st, TI2nd, and TI3rd groups. The dominant species in TI1st were *Klebsiella*, *Blautia*, and *Veillonella*. *Enterobacteriaceae* and *Proteobacteria* were the biomarkers in the TI3rd group. Recent research showed that *Klebsiella* activates dendritic and epithelial cells *via* Toll-like receptors, initiating the production of interleukin 18 to recruit and activate Th1 cells, triggering an inflammatory response (Atarashi et al., 2017). *Blautia* can promote short-chain fatty acids, thereby resisting inflammation and maintaining intestinal homeostasis (Scheiman et al., 2019). *Veillonella* converts the lactic acid produced during exercise into short-chain fatty acid propionate to improve muscle health and exercise capacity (Scheiman et al., 2019). In agreement with previous research (Li et al., 2016b), individuals living in the plateau environment require much energy to maintain a healthy metabolism; therefore, the abundance of bacteria that promote short-chain fatty acids and energy absorption may be higher. The *Enterobacteriaceae* was associated with intestinal microbiota imbalances. The dietary and living environment modification had a greater impact on *Enterobacteriaceae.* A previous study found that after Danes travel to India, their intestines are colonized by Enterobacteriaceae bacteria, resulting from eating habits and living environment changes (Dall et al., 2019). The increase in oxygen content of the air was a critical factor in the significant rise of the relative abundance of *Proteobacteria* phylum (Shin, Whon & Bae, 2015). Besides, *Enterobacteriaceae* belongs to the *Proteobacteria* phylum. In comparison with Tibet, the oxygen content in Xianyang was higher, which might cause a substantial increase in the abundance of the *Proteobacteria* and *Enterobacteriaceae* following migration (Shin, Whon & Bae, 2015).

### Variations in enterotypes and pathways of Tibetan women intestinal microbiota following migration

Enterotype was defined as a “densely populated area in a multidimensional space of community composition” and is not affected by other factors, such as age, gender, cultural background, and geographic location (Costea et al., 2018). Our study found that the proportion of ET_B was higher in the HI group, and the proportion of ET_P in the TI1st group was higher, but there was no significant difference. The B/P of the HI group was significantly higher than that of the TI1st group. Previous studies reported that most Han populations have ET_B (Nakayama, Zhang & Lee, 2017). However, the enterotype is affected by the genetic background of the host. The enterotype of the Mongolian population, whose diet is similar to that of the Tibetans, is dominated by the ET_P (Nakayama, Zhang & Lee, 2017). *Prevotella* dominates the enterotype of the ET_ P. Our previous research also found that due to the influence of temperature and oxygen, the abundance of *Prevotella* increase with altitude (Liu et al., 2021). Furet et al. found that B/P is negatively correlated with energy intake, and Tibetans have higher meat intake (Furet et al., 2010). Other studies have also found that Tibetans have higher energy intakes (13.7MJ/d) than the Han population (11.1 MJ/d) (Ge, Zhai & Wang, 1997). The plateau environment is a great challenge to human survival. We speculate that it may be due to Tibetan women’s need to consume more high-calorie protein foods to maintain basic metabolism and everyday activities, so the B/P in their intestines is low. Our research found no significant change in the enterotype and B/P after Tibetan women migrated to the plains for ten months. We speculate that it may be due to the high diversity of Tibetan women’s intestinal microbiota and the relatively stable community structure, which can resist changes in the environment and diet.

We found that the abundance of some pathways was up- or down-regulated during the migration of Tibetan women. BCAA (branched-chain amino acids) are mainly composed of leucine, valine, and isoleucine, associated with type 2 diabetes and insulin resistance (White & Newgard, 2019). The decrease in protein intake caused a decline in the amine transport pathway, and the functional gene pathways involved in alanine, aspartate, and glutamate metabolism, in addition to lysine biosynthesis, were downregulated. Tryptophan metabolism plays a vital role in maintaining intestinal immune balance (Roager & Licht, 2018; Spencer, Fragiadakis & Sonnenburg, 2019; Agus, Planchais & Sokol, 2018). However, we compared the gene pathways of TI1st, TI2nd, and TI3rd groups and found that the gene pathways of the Tibetan women’s intestinal microbiota did not significantly change after migration to Xianyang. No changes in intestinal microbiota gene pathways were found after comparing TI1st and TI3rd. The results indicate that the gene pathways of the intestinal microbiota did not significantly change after Tibetan women migrated to Xianyang for ten months. Moreover, the abundances of amino acid-related enzymes, DNA repair and recombination proteins, DNA replication proteins, ribosomal biogenesis, transcription factors and transporters between the HI and TI1st groups were significantly different. When comparing the gene pathways of HI and TI3rd groups, there were significant differences in the abundance of amino acid-related enzymes, DNA repair and recombination proteins, ribosomal biogenesis, transcription factors, and transporters similar to the result of comparison between HI and TI1st. Therefore, we speculate that Tibetan women’s intestinal microbiota was less affected by changes in the living environment and dietary habits (TI2nd and TI3rd).

## Conclusions

Our study demonstrated significant differences in the diversities and compositions of intestinal microbiota between Tibetan women and Han women. However, the intestinal microbiota diversity, structure, and gene pathways of Tibetan women who migrated to Xianyang for one day, six months, and ten months were similar. The time length of migration did not affect the intestinal microbiota comparison between Han and Tibetan women, indicating that Tibetan women’s intestinal microbiota was less affected by migration. Therefore, the present study supports the hypothesis that the intestinal microbiota of Tibetan women has a certain degree of stability due to many factors, creating resistance to the influence of the living environment and dietary habit changes caused by migration. Although the research was subjected to multiple limitations, such as low subject number among Tibetan women, especially lack of male volunteers, the results emphasize the importance of more research to improve our understanding of the link between migration and gut microbiota. Future research should use genome-wide association studies and metagenomics to explore the relationship between host genetics and intestinal microbiota and should use conventionalized germ-free mice to verify metabolomics further.

## Supplemental Information

10.7717/peerj.12036/supp-1 Supplemental Information 1 Supplementary Figures and Tables

10.7717/peerj.12036/supp-2 Supplemental Information 2 Comparison of alpha diversity of intestinal microbiota between Tibetan males and females

10.7717/peerj.12036/supp-3 Supplemental Information 3 Comparison of beta diversity of intestinal microbiota between Tibetan males and females

10.7717/peerj.12036/supp-4 Supplemental Information 4 Comparison of bacterial abundance between Tibetan males and females at the phylum level (A) and genus level (B)

10.7717/peerj.12036/supp-5 Supplemental Information 5 LDA values of Fig. 1 and 4B

10.7717/peerj.12036/supp-6 Supplemental Information 6 16S rRNA gene V3-V4 region Illumina MiSeq sequencing platform statistics

10.7717/peerj.12036/supp-7 Supplemental Information 7 Collection information of stool samples

10.7717/peerj.12036/supp-8 Supplemental Information 8 Stool samples information of Tibetan and Han population

10.7717/peerj.12036/supp-9 Supplemental Information 9 Comparison of bacterial abundance between Tibetan males and females at the phylum and genus levels

10.7717/peerj.12036/supp-10 Supplemental Information 10 Comparison of bacterial abundance between TS1st, TS2nd and TS3rd groups at genus level

10.7717/peerj.12036/supp-11 Supplemental Information 11 Raw data in Fig. 1 and 3

10.7717/peerj.12036/supp-12 Supplemental Information 12 Chinese questionnaire

10.7717/peerj.12036/supp-13 Supplemental Information 13 Questionnaire

## Additional Information and Declarations

### Data Availability

The following information was supplied regarding data availability: The raw sequence data are available at the Genome Sequence Archive at the Data Center, Beijing Institute of Genomics (BIG), Chinese Academy of Sciences: CRA002412 and CRA002780.
https://bigd.big.ac.cn/bioproject/browse/PRJCA002354

https://bigd.big.ac.cn/bioproject/browse/PRJCA002832.
