# Supplementary material for: Migration effects on the intestinal microbiota of Tibetans
Source: PeerJ. 2021 Oct 18;9:e12036. doi: 10.7717/peerj.12036 (PMC8530097; doi:10.7717/peerj.12036)
Supplement: Supplemental Information 1 [file peerj-09-12036-s001.docx]

Supplementary Material

## Supplementary Figures

##
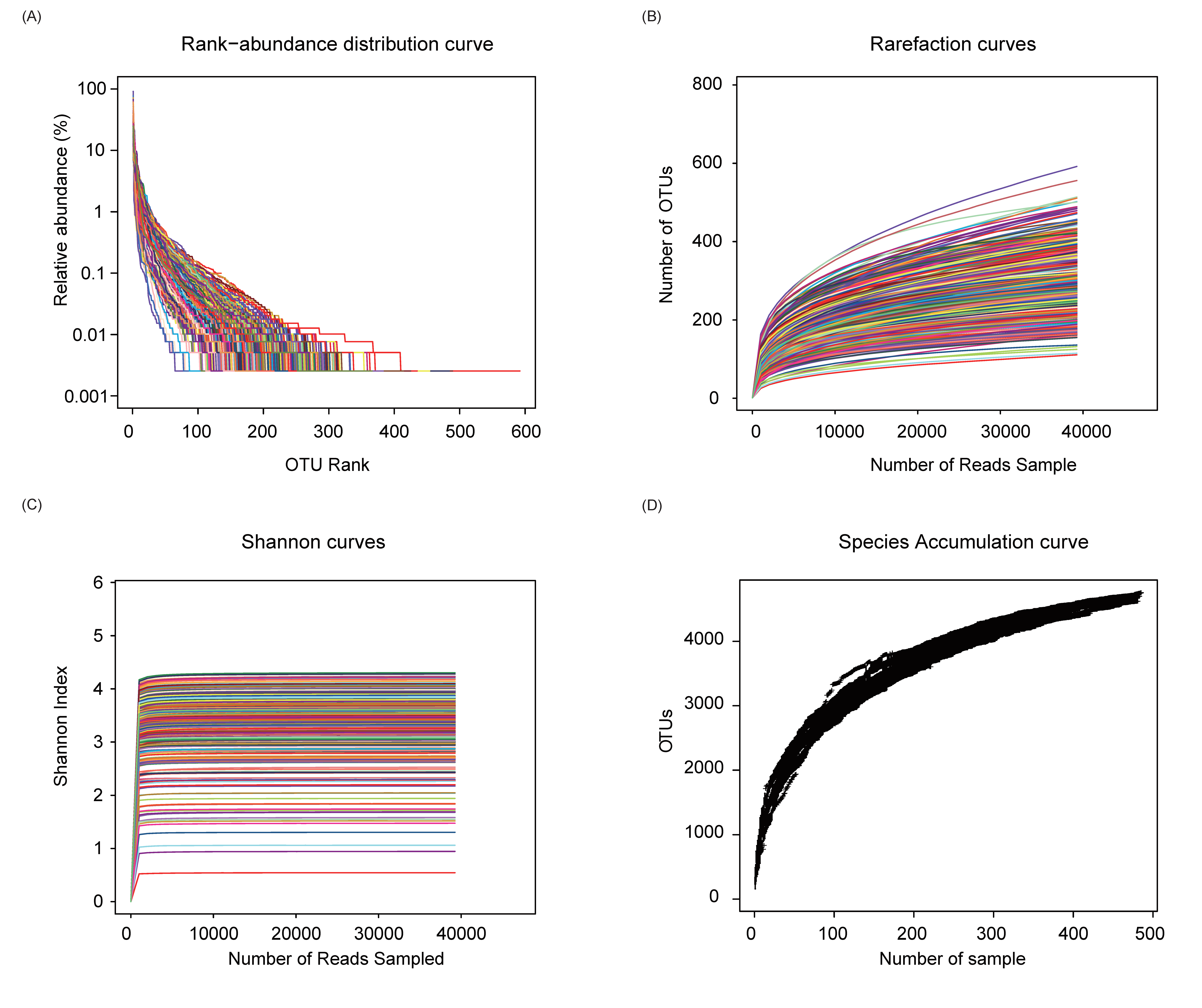


**Figure S1** Quality assessment. (A) Rank−abundance distribution curve analyses of all samples. (B) Rarefaction curve analyses of all samples. (C) Shannon curves analyses of all samples. (D) Species accumulation curve.


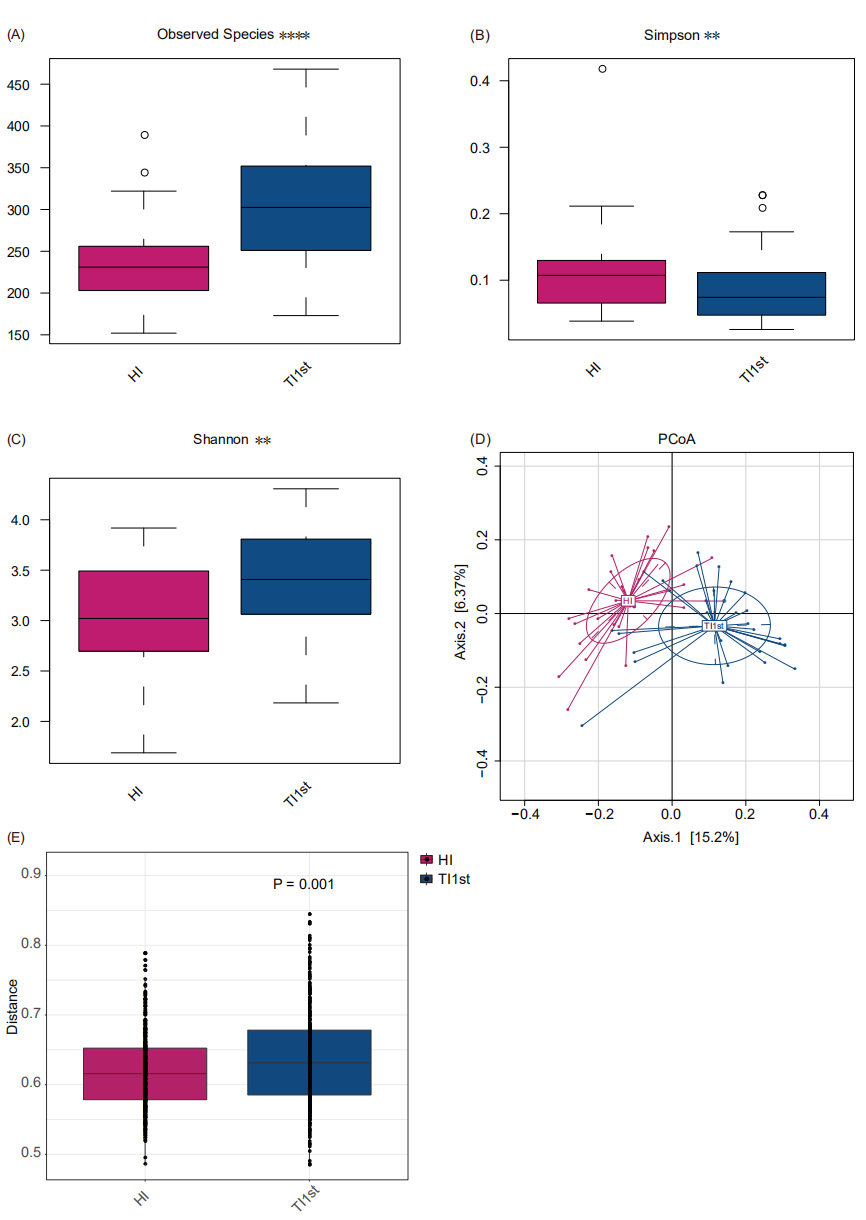


**Figure S2** Comparison of alpha and beta diversity of intestinal microbiota between Tibetans and Han population. (A) Composition of bacterial communities. The alpha diversity (B) and beta diversity (unweighted UniFrac distance) (C-D) of the intestinal microbiota of Tibetans are significantly higher than that of Han population, ***P* < 0.01, *****P* < 0.001.

.
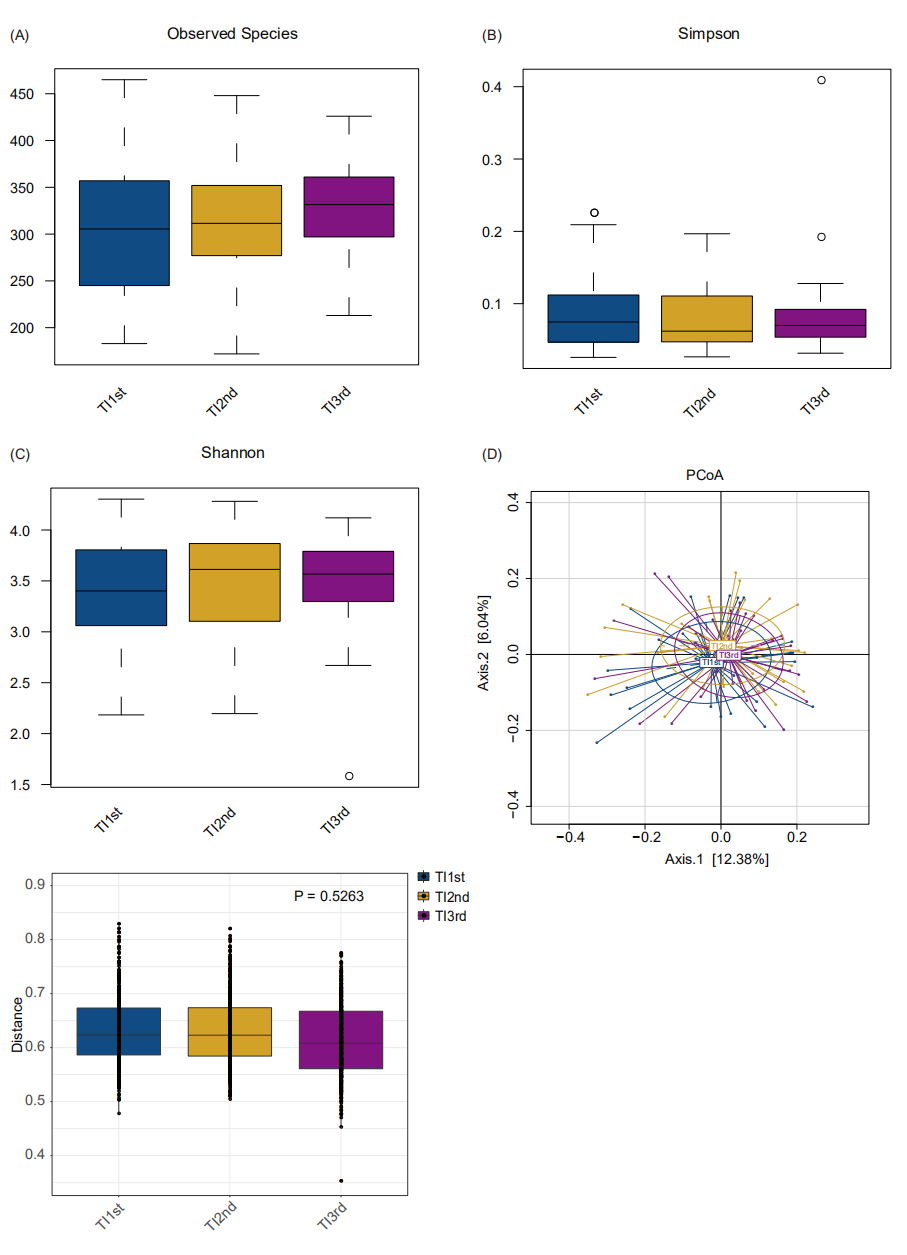


**Figure S3** Comparison of alpha and beta diversity of Tibetans intestinal microbiota at different migration times. (A-C) No significant difference in the alpha diversity (A-C) and beta diversity (unweighted UniFrac distance) (D-E) of the intestinal microbiota among the TI1, TI2nd, and TI3rd groups, *P* > 0.05.


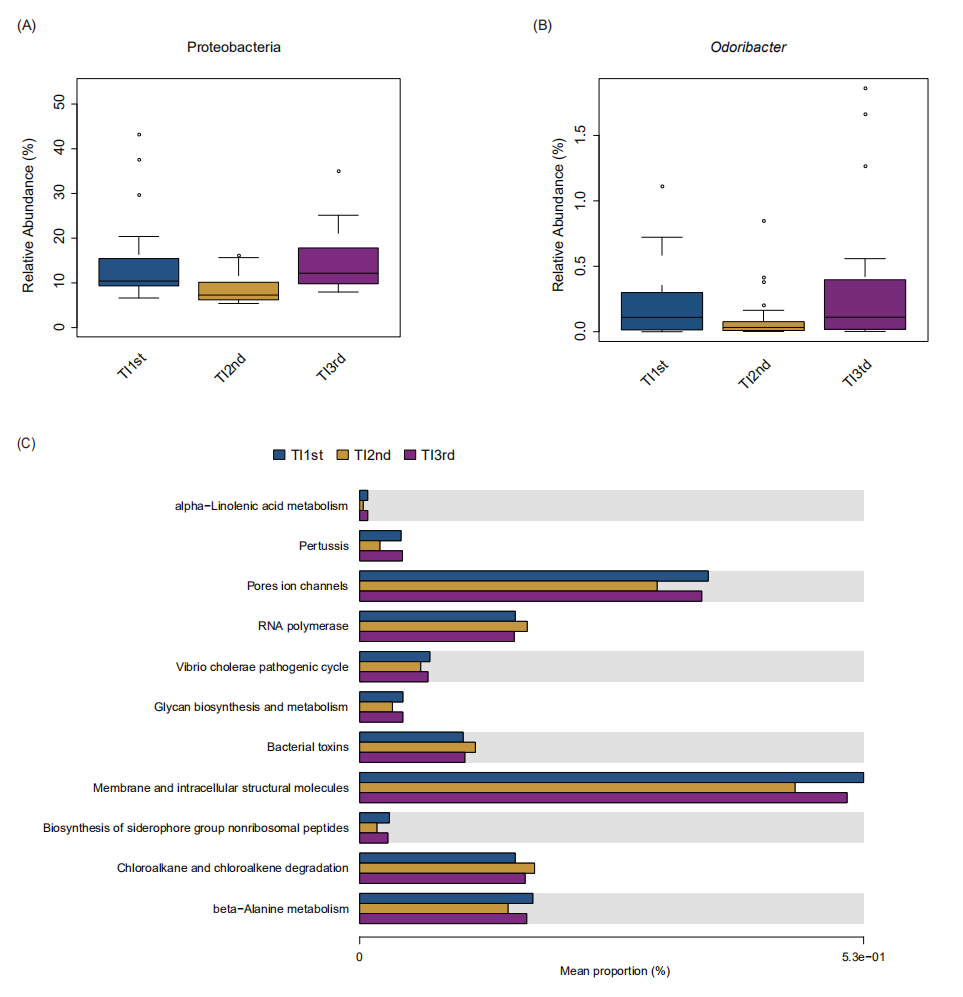


**Figure S4** Comparison of bacterial abundance and KEGG pathways of Tibetans intestinal microbiota at different migration times. No significant difference in bacterial abundance in the TI1st, TI2nd, and TI3rd groups at the phylum level (A) and the genus level (B), *P* > 0.05. (C) No significant difference in the KEGG pathway of TI1st, TI2nd, and TI3rd groups.

## Supplementary Tables

**Supplementary Table S1** The different KEGG pathways between the TI1st and TI3rd groups.

| KEGG pathays | TI1st (mean) | TI3rd (mean) | *P* value |
| --- | --- | --- | --- |
| Proximal tubule bicarbonate reclamation | 0.023 | 0.029 | 0.060 |
| Chloroalkane and chloroalkene degradation | 0.165 | 0.175 | 0.066 |
| Cell division | 0.068 | 0.063 | 0.070 |
| Flavonoid biosynthesis | 0.005 | 0.007 | 0.079 |
| Ether lipid metabolism | 0.002 | 0.002 | 0.099 |
