# Supplementary figures and images for: Migration effects on the intestinal microbiota of Tibetans
Source: PeerJ. 2021 Oct 18;9:e12036. doi: 10.7717/peerj.12036 (PMC8530097; doi:10.7717/peerj.12036)

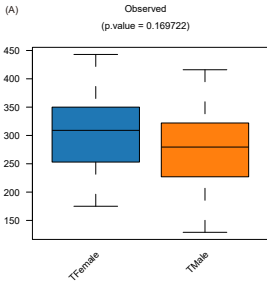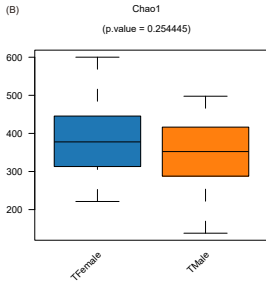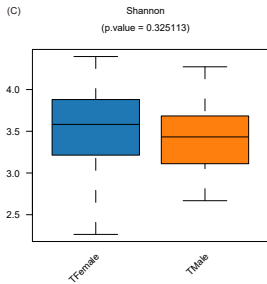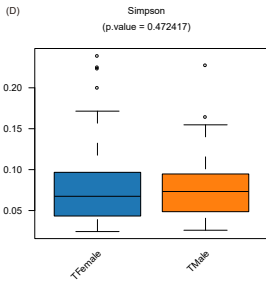

Supplement: Supplemental Information 2 [file peerj-09-12036-s002.pdf]

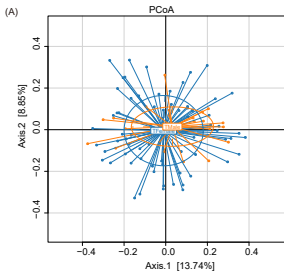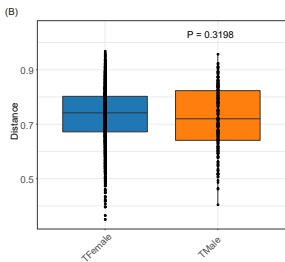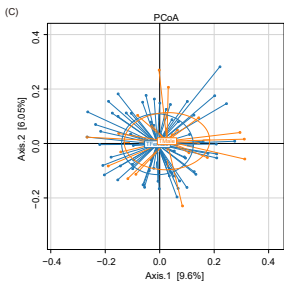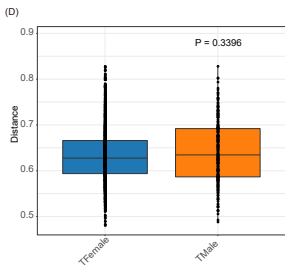

Supplement: Supplemental Information 3 [file peerj-09-12036-s003.pdf]

(A)

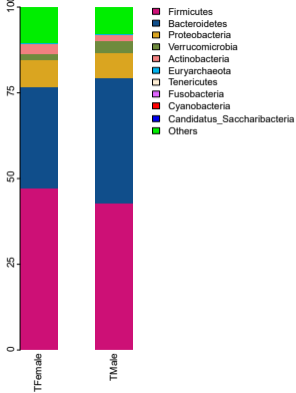

(B)

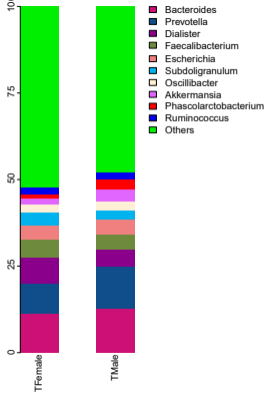

Supplement: Supplemental Information 4 [file peerj-09-12036-s004.pdf]
